# Comparison of Indoor Air Quality in Summer and Winter According to Four-Way Cassette Fan Coil Unit Operation in a Four-Bed Ward

**DOI:** 10.3390/toxics10090504

**Published:** 2022-08-28

**Authors:** Jungsuk Lee, Ik-Hyun An, Su-Hoon Park, Kyung-Rae Lee, Young-Won Kim, Se-Jin Yook

**Affiliations:** 1 School of Mechanical Engineering, Hanyang University, Seoul 04763, Korea; 2 Green Energy & Nano Technology R&D Group, Korea Institute of Industrial Technology, Gwangju 61012, Korea

**Keywords:** indoor air quality, age of air, fan coil unit, ventilation system, air cleaner

## Abstract

This study targeted a four-bed ward with a ventilation system and a four-way cassette fan coil unit (4-way FCU) installed on the ceiling. The indoor air quality under summer and winter conditions was comparatively analyzed. The age of air was calculated by conducting tests and simulations under diverse conditions, assuming that the ventilation system and 4-way FCU were continuously operating. The use of an air cleaner and ward curtain was investigated for its impact on the air quality in the breathing zone of a patient lying on the bed, and effects of the airflow and discharge angle of the 4-way FCU were considered. Because the 4-way FCU was installed in the central part of the ceiling, where indoor air is sucked in and subsequently discharged in four directions, the age of air at each bed was found to vary depending on the airflow and discharge angle of the 4-way FCU. When the airflow and discharge angle of the 4-way FCU was fixed, the age of air at each bed appeared to be lower during winter heating than in summer cooling mode. The age of air was significantly lowered at each bed, depending on the use of the curtain and the air cleaner along with the ventilation system and 4-way FCU, and appropriate seasonal operating conditions were identified to maintain a lower age of air at each bed.

## 1. Introduction

The recent COVID-19 global epidemic caused rampant infection, which led to increased interest in the spread of viruses. The COVID-19 pandemic has made people more active indoors and led to many cases of infection in confined indoor spaces [1,2,3,4,5]. Virus infection transmission is largely divided into droplet infection and airborne infection [6]. In droplet infection, the virus can spread to nearby people through coughing or sneezing, generally occurring when the particle size is 5 μm or larger. In contrast, airborne infection is caused by particles including viruses, generated from sneezing or breath flow along air currents, and can cause infection in an unspecified number of people at a distance; the particle size here is chiefly smaller than 5 μm. In the case of COVID-19, the size of the virus particle was found to range between 0.06–0.14 μm [7]. Van Doremalen et al. [8] conducted a study on the survival time of coronavirus, and it was reported that the virus can survive for up to three hours in the form of an aerosol. Therefore, it is highly likely that the COVID-19 virus is transmitted via airborne infection. Additionally, the possibility of airborne COVID-19 infection has recently been recognized [9].

Airborne infection refers to transmission by airflow when droplets generated through coughing or sneezing become small-sized particles by evaporation. Fears et al. [10] showed that coronavirus can be particularized, and confirmed that the sprayed virus particles can be suspended for an extended period of time. Setti et al. [11] identified movement of the COVID-19 virus in the atmosphere, and recognized its association with a high concentration of Particulate Matter less than 10 μm (PM_10_). Yao et al. [12] investigated the relationship between PM concentrations and mortality of COVID-19. They indicated that higher PM_2.5_ and PM_10_ concentrations were related to higher COVID-19 mortality rates. Somsen et al. [13] conducted an experiment to determine how long droplets emitted through coughing stayed in an experimental chamber equipped with ventilation facilities. It was confirmed that lower levels of ventilation led to failure in quickly eliminating droplets and to their persistence indoors. Most studies found that the spread of coronavirus is highly correlated with PM concentration levels, which means that poor indoor ventilation can increase the likelihood of the virus spreading.

Elsaid and Ahmed [14] suggested using ventilation systems equipped with high-efficiency particulate air (HEPA) filters, continuously supplying clean air into air-conditioned places to limit the propagation of COVID-19 viruses. Srivastava et al. [15] considered a situation where an infected person was in an office building and reported that increasing the ventilation flow rate was helpful for reducing the infection risk indoors. Dai and Zhao [16] investigated the relationship between ventilation rate and infection probability and suggested ensuring a sufficient ventilation rate per infected person. Therefore, to control the spread of coronavirus, indoor ventilation is essential, and there have been several studies to address this. Noh et al. [17] studied the effect of using a ventilation system and air cleaner on indoor air quality in a laboratory simulation of a four-bed ward. It was found that indoor air quality significantly improved when ventilation systems and air cleaners were simultaneously utilized. Noh and Yook [18] observed changes in indoor air quality depending on the number of air cleaners operating in school classrooms. They confirmed that particles could be quickly removed from rooms when multiple air cleaners were operating. Casagrande and Piller [19] conducted a study on the removal of contaminants using a laminar airflow ventilation system attached to the ceiling of an operating room, and a portable laminar airflow system. Consequently, they identified different levels of removed contaminants depending on flow patterns and the amount of ventilation. Liu and Lin [20] studied air quality in a bedroom by attaching a ventilation system around the bed, and confirmed that higher ventilation flow resulted in lower age of air. Yin et al. [21] evaluated ventilation performance in a situation where a person was lying on a bed in a test chamber, and confirmed that the air quality can be determined by the ventilation flow rate and air temperature. Hormigos-Jimenez et al. [22] experimented by altering the furniture arrangements in a test chamber. Their findings indicated that the age of air varied based on the furniture arrangement under the same flow rate conditions. Akbari and Salmanzadeh [23] studied indoor air quality based on ventilation and the operation of an air cleaner in an office. The age of air differed depending on the ventilation method and position of the air cleaner. By numerical analysis, Rabanillo-Herrero et al. [24] studied ventilation efficiency in an L-shaped room and confirmed that ventilation efficiency can vary depending on location of air inlet and outlet. Navaratnam et al. [25] reviewed the latest engineering control preventive measures for reducing the spread of COVID-19 viruses, and suggested installing ventilation systems with ultraviolet germicidal irradiation and bipolar ionization.

It is necessary to operate an air conditioner to maintain proper indoor temperature, and its contribution has been underpinned by many studies. Meiss et al. [26] confirmed that flow pattern can vary indoors depending on the temperature of the inflowing air from outside according to season. According to Ishiguro et al. [27], flow patterns and temperature distributions may differ due to the flow rate of ceiling air-conditioners in offices. Kato and Yang [28] studied ventilation effects with the simultaneous operation of a wall-mounted air conditioner and air cleaner in an office. They confirmed that ventilation efficiency was affected by the area of the air cleaner outlet. Lee et al. [29] conducted a study on the effect of the louver angle and attachment location of a wall-mounted air-conditioner in a test chamber, and confirmed that the age of air indoors differed based on the louver angle. Most studies relating to usage of air-conditioners to maintain appropriate indoor temperature have focused only on the effects of air conditioners; studies of indoor air quality relating to the simultaneous operation of flow generators other than air conditioners were found to be insufficient.

It is crucial to purify the air in a ward with immunocompromised patients. Particularly, operation of the air-conditioning system is essential to maintain the body temperatures of these patients. A ventilation system and portable air cleaner can be used in hospital rooms for air purification. Recently, four-way ceiling fan coil units (4-way FCU) have been widely used for cooling and heating hospital rooms. However, there has been insufficient research on the relationship between each airflow generator in cases where diffierent airflow generators, such as fan coil units, ventilation systems, and air cleaners, are used simultaneously in hospital rooms. Since the operating conditions of cooling and heating in hospital rooms may vary seasonally, it is necessary to analyze indoor air quality according to the seasonal operating conditions of the 4-way FCU and air cleaner. In this study, particle-removal experiments and numerical analysis were conducted to calculate and compare the age of air in a four-bed ward. Appropriate conditions for operating the 4-way FCU were analyzed in terms of indoor air quality, and in relation to the effects of ventilation systems, air cleaners, curtains, etc. in a four-bed hospital ward.

## 2. Materials and Methods

### 2.1. Description of Ward

**Figure 1:**
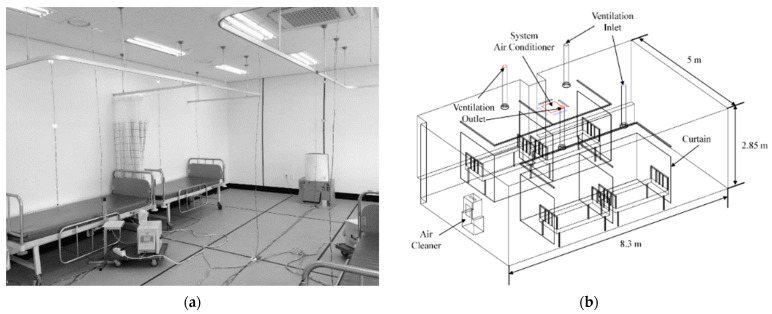

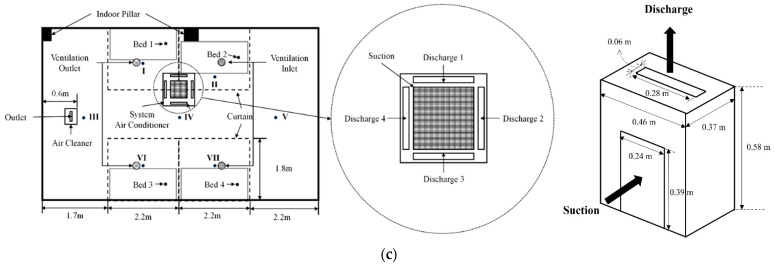
Composition of the four-bed ward laboratory: (**a**) laboratory photograph; (**b**) isometric view; (**c**) floor plan.

Figure 1 shows the spatial structure that simulated the four-bed ward. The room size was 8.5 × 5 × 2.85 m, and the room space volume was 118.3 m^3^. Four hospital beds were placed near the wall in the room, and one bed (Bed 2) was separated from the wall by the building column. The extent of separation was equal to the thickness of the column. Curtains were installed for each bed so that the space could be divided independently as needed. The ventilation system was connected to two air inlets and two outlets.Air inlets were installed on the ceiling of the areas with Beds 2 and 4, respectively, and outlets were installed on the ceiling of the areas with Beds 1 and 3, respectively. One four-way cassette fan coil unit (4-way FCU) was installed at a location close to the center of the ceiling, slightly tilted toward Beds 1 and 2. An air cleaner (0.46 × 0.37 × 0.58 m) was placed at a height of 0.5 m from the floor and installed 0.6 m away from the center of one wall, near to the ventilation outlet. The air cleaner sucked the air from its rear side and discharged it from the top.

### 2.2. Experimental Method

Experiments were conducted for two seasonal situations (summer and winter) to understand the effect of the 4-way FCU on indoor air quality according to season. The temperature by height was measured using a total of 21 T-type thermocouples at various locations in the room. The temperature measurement results were secured using data collection devices (i.e., NI cDAQ-9178 and NI 9213, National Instruments, Austin, TX, USA). The flow rates of various airflow generators were measured using a volume flow hood (Model testo 420, Testo, Inc., Titisee-Neustadt, Baden-Württemberg, Germany). Table 1 and Table 2 present the experimental conditions when operating the cooling function of the 4-way FCU in summer and those when operating the heating function of the 4-way FCU in winter, respectively. The ventilation system and 4-way FCU were always in operation. Based on the operation of air cleaners, use of curtains, flow rate, and discharge angle for the 4-way FCU, 16 cases were set for each season, and the experiment was conducted. The total flow rate of the ventilation system was 534 CMH. The temperature range of air inflow to the room through the ventilation inlet was 28–32 °C in summer, and 13–17 °C in winter. Total flow rate of the 4-way FCU was 584 and 706 CMH in low and high conditions, respectively. The 4-way FCU recirculated the room’s air. The temperature of air discharged from the 4-way FCU was 15.5 and 40 °C in summer and winter, respectively.

The high value of the angle between the ceiling and the air outlet of the 4-way FCU was set to 60°, so that air discharged from the 4-way FCU reached the hospital bed directly. The lower values of discharge angle for the 4-way FCU in summer and winter were set to 30° and 45°, respectively. This was done considering that cold exhaust air adequately reached the lower part of the room in summer, while hot exhaust air did not reach the lower part well in winter due to the density difference. The flow rate of the air cleaner was 288 CMH.

**Figure 2:**
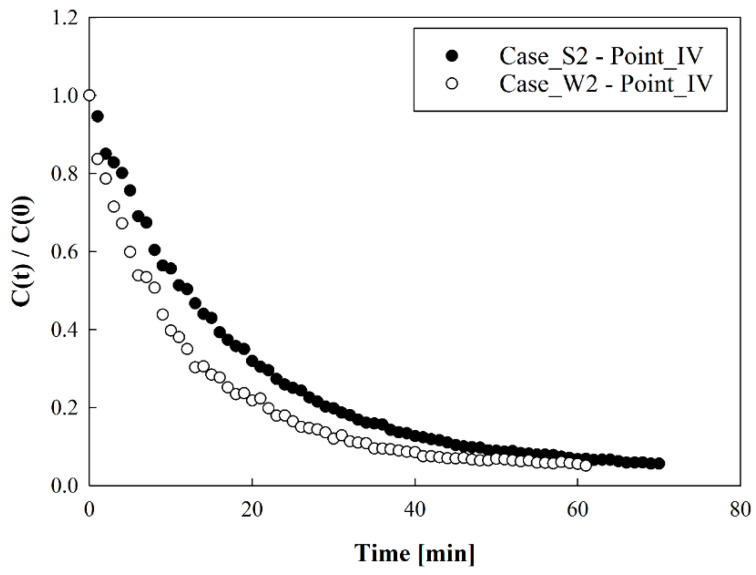
One of the measurement results for particle number concentration over time.

The method of Noh et al. [17] was introduced to determine the age of air by using aerosols. As shown in Figure 1, a condensation particle counter (CPC; Model CPC-0701, HCT Co., Ltd., Korea) was employed to measure particle number concentrations at seven locations, indicated from I–VII. After burning incense in a confined room, a fan was used for 15 min so that the particles from the incense were evenly distributed throughout the room. Subsequently, the fan was turned off and 15 min elapsed until indoor particle number concentration values were stabilized. Then, the ventilation system, air cleaner, and 4-way FCU were operated simultaneously. Figure 2 indicates an example of the particle number concentration measurement result, and shows the temporal changes of the particle number concentrations measured at position IV under the conditions of Cases S2 and W2. Here, the *y*-axis value represents the normalized particle number concentration using the particle number concentration value when the indoor flow was stabilized (time = 0 min) through the operation of various flow generators. As illustrated in Figure 2, the particle number concentration at each measurement location showed an exponential decrease. Therefore, the temporal change in particle number concentration was expressed using the following equation [17]:

(1) $$C=C_{0}+A\exp\left(-\frac{t}{\tau }\right)$$

here, *C* is the particle number concentration, *C*_0_ is the convergence value of the particle number concentration after a long time, *A* is the initial particle number concentration, *t* is the measurement time, and *τ* is the age of air obtained from the experimental results.

### 2.3. Numerical Method

ANSYS FLUENT *Release 16.1*, a commercial CFD code, was utilized to simulate the flow, temperature, and age of air for the space indicated in Figure 1. The flow was assumed to be steady-state, incompressible, and turbulent, and the *k-ε* realizable model was utilized for turbulence analysis [19,21]. For each flow generator, a velocity inlet condition was set at a position for discharging air into the room, a pressure outlet condition was applied to the position where air was sucked from the room, and the mass flow rate of the sucked air was equalized to that at the outlet. Meanwhile, no-slip conditions were set for all the walls. All walls in the ward were assumed to be at a uniform temperature. The indoor wall and air temperatures were set to 30 and 15 °C in summer and winter, respectively. The temperature of air discharged from the ventilation inlet and air cleaner into the room was set to 30 and 15 °C in summer and winter, respectively. The temperature of air discharged from the 4-way FCU was set to 15.5 and 40 °C in summer and winter, respectively. To calculate the age of air using CFD, the following equations were solved [30]:

(2) $$\frac{\partial }{\partial x_{i}}\rho u_{i}\Phi -\dot{J}\frac{\partial \Phi }{\partial x_{i}}=\rho$$

(3) $$\dot{J}=-\left(\rho D_{m}+\rho D_{t}\right)\frac{\partial \Phi }{\partial x_{i}}$$

here, Φ indicates the age of air, *u_i_* is the flow velocity, *ρ* is the air density, *j* is the diffusion term, and *D_m_* and *D_t_* indicate molecular diffusivity and turbulent diffusivity, respectively. The age of air at the outlets of the ventilation system and air cleaner was set to 0 considering that the ventilation system and air cleaner were equipped with HEPA filters. Given that the filter used in the 4-way FCU had very low collection efficiency, the age of air at the outlet of the 4-way FCU was set to be the same as the average age of air introduced through the inlet.

## 3. Results and Discussion

**Figure 3:**
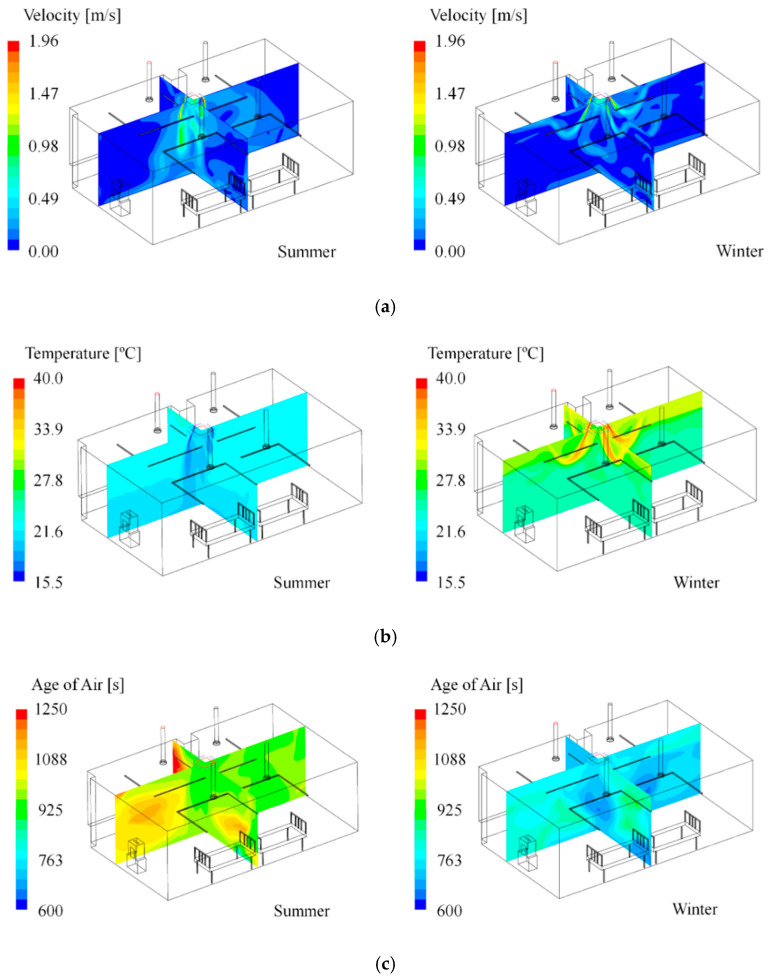
One of the simulation results (**left**: Case S2 in summer, **right**: Case W2 in winter); (**a**) velocity contour; (**b**) temperature contour; (**c**) age of air contour.

Figure 3 exemplifies the distribution of flow velocity, temperature, and age of air predicted by simulation; the left side exhibits the result of Case S2 in summer, and the right shows the outcome of Case W2 in winter. As shown in Figure 3a, in summer, cold air from the 4-way FCU reaches the floor adequately. In contrast, during winter, warm air from the 4-way FCU does not reach the floor, but rises again at a height of about 1.6 m. This is because the flow pattern can be changed under the influence of a heat source, due to the thermal buoyancy effect [31,32,33]. Accordingly, the predicted indoor temperature in winter varied significantly based on height, while the indoor temperature in summer appeared relatively even (Figure 3b). The analysis results of the age of air presented in Figure 3c confirmed that the average value of the age of air at the inlet of the 4-way FCU was well reflected at the outlet. Hence, the age of air varied depending on the cooling and heating operational conditions according to season.

**Figure 4:**
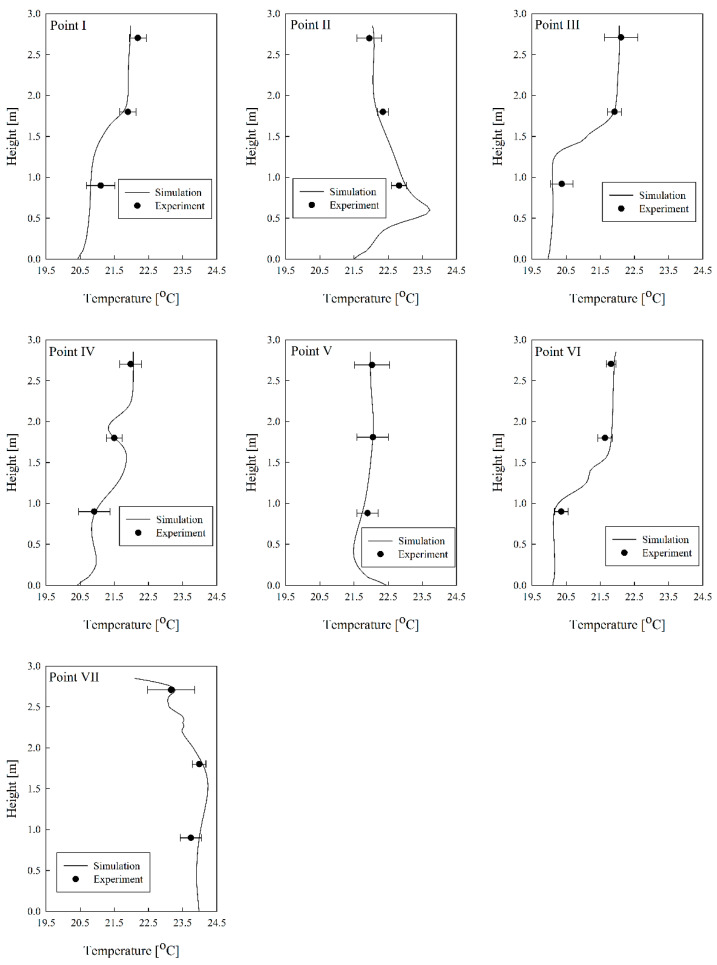
Comparison of experimental and simulation results for temperature distribution by height at seven locations in laboratory.

**Figure 5:**
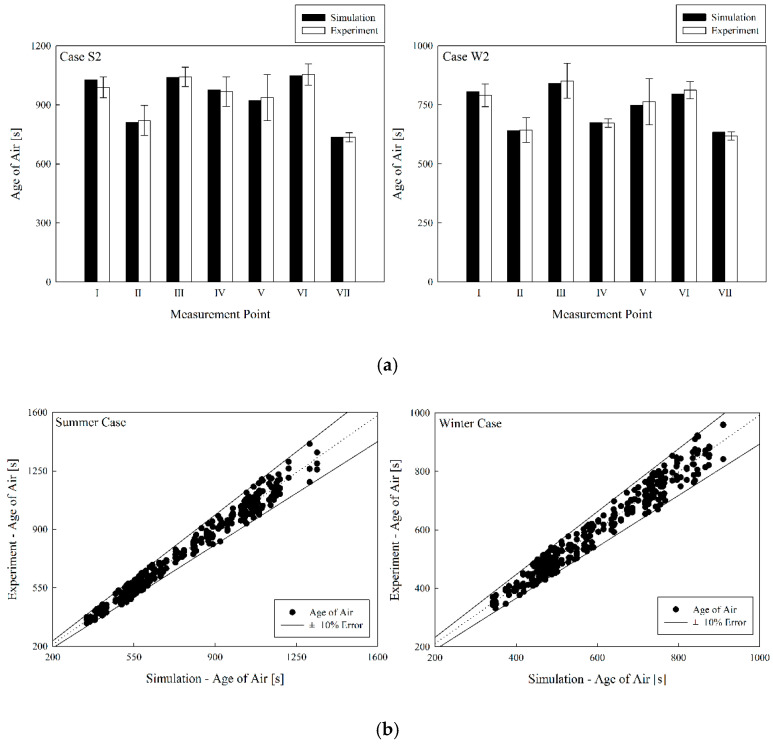
Comparison of age of air between experiment and simulation; (**a**) comparison of Cases S2 and W2; (**b**) comparison of all cases in summer and winter.

Figure 4 shows the comparison between the experiment and simulation of the temperature distributions for Case S2 in summer. It was found that the temperature distribution by height at locations I–VII was highly consistent. Meanwhile, Figure 5a shows the comparison between experimental and simulation age of air at a height of 0.85 m from the floor, at locations I–VII for Case S2 in summer and Case W2 in winter, and confirms that they were highly consistent. In both summer and winter, the age of air was found to be low at locations II and VII, where clean air was directly introduced from the ventilation inlet. Figure 5b presents the comparison results for the age-of-air values at seven locations for all cases in Table 1 and Table 2 between the experiment and the simulation; the error was within ±10% and had a good accord. Thus, the simulation method applied in this study has a confirmed high prediction accuracy.

Because this study focuses on the age of air in a four-bed ward, it was assumed that patients were lying on each bed. Thus, the age of air in the patients’ breathing zone was analyzed. Since the previous verification results confirmed that the prediction accuracy of the simulation was high, the simulation results were compared and analyzed. It was assumed that the ventilation system and 4-way FCU were always in operation, and a comparative analysis on the age of air was conducted based on the usage status of curtain and air cleaner and the operation conditions of the 4-way FCU.

**Figure 6:**
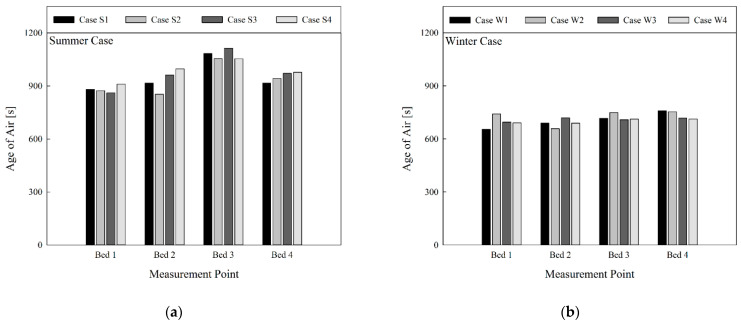
Comparison of age of air at bed location by season (in cases of ventilation system operation, four-way cassette fan coil unit operation, non-operation of air cleaner, no use of curtain): (**a**) summer; (**b**) winter.

Figure 6a shows a comparison of the age of air in summer when the air cleaner was not in operation and the curtain was folded; Figure 6b presents the comparison of the age of air in winter. There was a change in the age of air per bed based on the flow rate and discharge angle of the 4-way FCU. However, it was difficult to find a general tendency. In the summer cooling mode, the age of air at Bed 3 was higher than for the other beds. This was largely attributable to the location of Bed 3, which was closer to one of the ventilation outlets. Bed 1 was also located closer to another ventilation outlet, but the age of air at this location was lower than that of Bed 3, because Bed 1 was located closer to the 4-way FCU, so relatively cleaner air from the 4-way FCU reached the location. Although Beds 2 and 4 were closer to ventilation inlets, they showed similar values for age of air to that of Bed 1. This is because the 4-way FCU was located between the ventilation inlets and outlets. Hence, air was spread to this area that had stayed indoors for a longer time than the air supplied from the ventilation inlets. Conversely, when comparing the age of air in summer and winter under the same conditions (i.e., S1 vs. W1, S2 vs. W2, etc.), the age of air appeared lower in winter than in summer, and similar age of air values were found regardless of bed locations. This is due to the presence of a large flow-circulation pattern throughout the entire room in summer, when the cold air from the 4-way FCU adequately reached the floor. In contrast, the warm air from the 4-way FCU did not reach the floor in winter and mainly circulated in the upper part of the room (due to the relatively rapid speed of airflows circulating in the upper part). Therefore, in winter, clean air from ventilation inlets was spread to each bed comparatively quickly. Since the 4-way FCU sucks the room’s air into its central intake and discharges the air through its four outlets, the 4-way FCU can expedite air circulation in the ward and as a result enhance air quality in regions where clean air from the ventilation inlets cannot be supplied efficiently. In the view of the air quality in the breathing zones of patients lying on the beds, this effect became more noticeable in the winter heating mode than in the summer cooling, due to faster air circulation in the upper part of the room caused by the thermal buoyancy effect.

**Figure 7:**
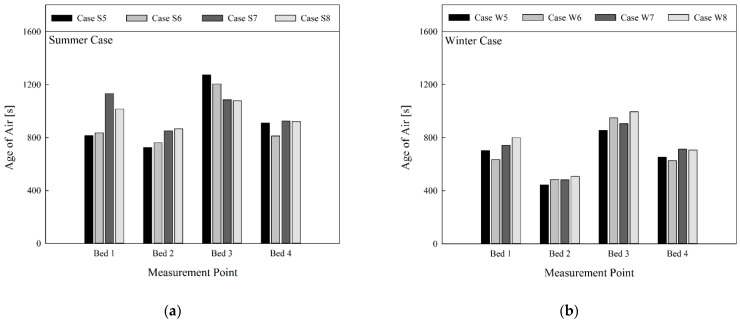
Comparison of age of air at bed location by season (in cases of ventilation system operation, four-way cassette fan coil unit operation, non-operation of air cleaner, and use of curtain): (**a**) summer; (**b**) winter.

Figure 7 gives comparison of the age of air when the curtains on all beds were fully unfolded, without operation of the air cleaner. Since each bed was placed in a relatively independent space due to the use of curtains, the age of air appeared lower in Beds 2 and 4 that were located under the ventilation inlets. In contrast, the age of air appeared higher in Beds 1 and 3, located below the ventilation outlets. The age of air appeared lower for Bed 2 than Bed 4, and lower for Bed 1 than Bed 3. This was because Beds 1 and 2 were located relatively closer to the 4-way FCU, so the air from the 4-way FCU spread faster in the area. There was no general tendency in the change of age of air in line with the flow rate of the 4-way FCU, but the age of air was generally lowered when the discharge angle was increased after fixing the flow rate of the 4-way FCU, except in the case of Bed 3 in summer. This is because when the air discharge angle of the 4-way FCU was larger (i.e., 60°), air from the 4-way FCU contributed to more active re-circulation of air in the relatively independent spaces of Beds 2 and 4, located below the ventilation inlets. This resulted in smoother inhalation of clean air from ventilation inlets into the inlet part of the 4-way FCU. Conversely, when the discharge angle of the 4-way FCU became smaller (i.e., 30° or 45°), air from the 4-way FCU was partly expelled into the space between the upper end of the curtain and the ceiling, which led to relatively slower re-circulation of air within the relatively independent spaces of Beds 2 and 4. On comparison under the same conditions (i.e., S5 vs. W5, S6 vs. W6, etc.) when the 4-way FCU was operating in heating mode in winter, the age of air appeared lower compared with cooling mode in summer. This is compliant with the previous results. When all curtains were fully unfolded in the four-bed ward equipped with the ventilation system (and without the air cleaner), adjustment of the discharge angle of the 4-way FCU is recommended such that the air from the 4-way FCU can directly reach the relatively independent spaces below the ventilation inlets. This helps clean air from the ventilation inlets to enter the 4-way FCU and be spread to the regions where the clean air cannot be supplied efficiently.

**Figure 8:**
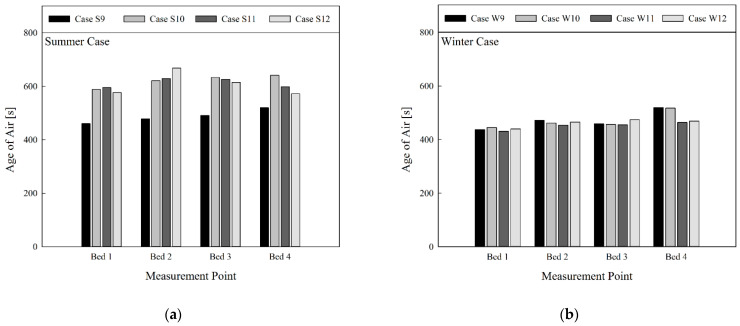
Comparison of age of air at bed location by season (in cases of ventilation system operation, four-way cassette fan coil unit operation, operation of air cleaner, and no use of curtain): (**a**) summer; (**b**) winter.

Figure 8 presents comparison of the age of air based on operating conditions of the 4-way FCU in a situation where the air cleaner was operated, and the curtain was folded. Compared with the results in Figure 6, where neither the air cleaner nor the curtain was used, the age of air was significantly lowered. This was because more clean air flowed into the room due to usage of the air cleaner. In addition, a similar level of age of air was found for all beds in each case. This is because clean air was more smoothly supplied to Bed 3 by operating the air cleaner near the ventilation outlets. As shown in Figure 8a, when the 4-way FCU was operating in the cooling mode in summer, Case S9 showed a significantly lower age of air compared to other cases for all beds. The reason for thise results is that the flow rate of the 4-way FCU was lower in Case S9, and the discharge angle was larger compared to other cases. Hence, the flow circulation in the central part of the room in which the beds were gathered became more active. In contrast, interference with the airflow from the air cleaner was reduced, and consequently air of a lower age could be inhaled into the 4-way FCU. Therefore, the age of air at the inlet part of the 4-way FCU was 482.5 s in Case S9, which was lower on average by 138 s compared to that of other cases. However, as shown in Figure 8b, when the 4-way FCU was operating in the heating mode in winter, the age of air was overall lower than in summer. This was already shown in the previous results. The age of air was found to be almost identical on comparison by bed location, except for W9 and W10 conditions for Bed 4. There was no significant difference, but among the winter conditions (W9–12), W11 showed a slightly lower average age of air. Operating an air cleaner in addition to the ventilation system can enhance overall indoor air quality. Moreover, in the view of the air quality in the breathing zones of patients lying on the beds with all curtains folded, the 4-way FCU can be operated under any conditions of flow rate and discharge angle in the winter heating mode, whereas it is recommended to operate the 4-way FCU under the conditions of low flow rate and large discharge angle in the summer cooling mode.

**Figure 9:**
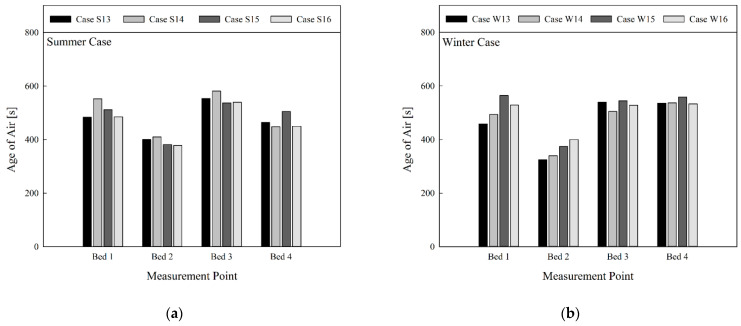
Comparison of age of air at bed location by season (in cases of ventilation system operation, four-way cassette fan coil unit operation, operation of air cleaner, and use of curtain): (**a**) summer; (**b**) winter.

Figure 9 shows comparison of the age of air based on operational conditions of the 4-way FCU in a situation where the air cleaner was operated, and curtains of all beds were unfolded. It was difficult to find a general tendency to describe the effect of the 4-way FCU’s flow rate or discharge angle on the age of air. Compared with the results of Figure 7 where the air cleaner was not in operation, the age of air appeared relatively lower at Beds 2 and 4 located closer to the ventilation inlets. However, the age of air appeared significantly lower for all beds due to the use of the air cleaner. Based on the results in Figure 9a, no significant difference was found when comparing the average age of air for each bed location in summer. However, the average value of age of air was found to be slightly lower under the conditions of S16. In contrast, when comparing the age of air between summer and winter under the same conditions (i.e., S13 vs. W13, S14 vs. W14, etc.), it can be found that the difference in age of air by season was slightly reduced, unlike in Figure 6, Figure 7 and Figure 8. This is because the clean air that was discharged upwards from the air cleaner moved in a desirable direction along the ceiling of the passage, connecting the positions III–IV–V after unfolding the curtain. The air was more effectively sucked by the 4-way FCU, and was well distributed indoors through the four outlets of the 4-way FCU. Figure 9b compares the average age of air for each bed location in winter, and reveals that the average age of air appeared slightly lower in the W13 condition. When the 4-way FCU, ventilation system, and air cleaner were simultaneously operating with all curtains unfolded, the air cleaner should be placed so that the clean air discharged from the air cleaner can be efficiently sucked into the 4-way FCU. There was little noticeable difference in the air quality in the breathing zones of patients lying on the beds, dependent on the flow rate and discharge angle of the 4-way FCU.

## 4. Conclusions

This study compared and analyzed the age of air at the respiratory position of patients who were assumed to be lying on beds in a four-bed ward. Analysis was carried out based on the cooling (summer) and heating (winter) modes of the 4-way FCU.

When only the ventilation system and the 4-way FCU were in operation and the curtains were folded, the following method was effective for improving the air quality at each bed: Clean air from the ventilation inlets moved adequately into the 4-way FCU, and subsequently reached the locations containing beds via the four outlets of the 4-way FCU. When the flow rate and discharge angle of the 4-way FCU were fixed, the age of air at the hospital beds was lower in the heating mode during winter than in the cooling mode during summer. Conversely, if the curtains were fully unfolded to create a somewhat independent space around each bed, the age of air appeared lower in areas where the beds were under ventilation inlets or closer to the 4-way FCU. There was no general tendency of change in the age of air in line with the flow rate of the 4-way FCU. However, Bed 3 was relatively far from the 4-way FCU and located under the ventilation outlet. Here, the age of air around the bed decreased as the discharge angle of the 4-way FCU increased; this was an exceptional case.

In contrast, it was confirmed that indoor air quality can be greatly improved by operating an air cleaner in a four-bed ward in addition to a ventilation system and 4-way FCU. Even when the air cleaner was operated simultaneously and the curtain was not used, the age of air at the hospital bed was lower in the winter heating mode than in the summer cooling mode, under conditions in which the flow rate and discharge angle of the 4-way FCU were fixed. In order to improve the air quality at each bed when the curtains were folded, it was helpful to decrease the flow rate and increase the discharge angle of the 4-way FCU in summer, and to decrease the flow rate and discharge angle in winter. In order to improve the air quality at hospital beds when the curtains were fully unfolded, a higher flow rate and lower discharge angle of the 4-way FCU were slightly effective in summer. In winter, a lower flow rate and higher discharge angle had minimal effectiveness, but there was no significant difference in terms of the average age of air at each bed.

It was found that the 4-way FCU is generally helpful for improving the air quality in regions where clean air from ventilation inlets cannot be supplied efficiently. In order to maximize the improvement of local air quality in a four-bed ward, the 4-way FCU needs to be operated so that clean air discharged from the ventilation inlets or the air cleaner can efficiently be sucked into the 4-way FCU. In addition, if the 4-way FCU operational conditions are controlled based on the results of this study, it can be expected that the air quality in the breathing zones of the patients lying on the beds can be well maintained at all times. It was confirmed that using an air cleaner in a ward with a ventilation system and 4-way FCU greatly contributed to improving the air quality in the ward. In general, the arrangements of ventilation inlets or outlets and a 4-way FCU in a four-bed ward are often very similar to the conditions set in this study. Furthermore, it is not easy to change the arrangement since these airflow generators are fixed to the ceiling. However, air cleaners can be utilized at any location, and so further research is encouraged on measures to improve more effectively the air quality around patient beds, based on the location of air cleaners and quantity of equipment in operation.

## Figures and Tables

**Table 1:** Cases for cooling mode of the four-way cassette fan coil unit in summer.

Summer Season Case					
Case	Ventilation System	Air Cleaner	Curtain	Four-Way Cassette Fan Coil Unit	
				Flow Discharge Angle	Flow Rate
S1	On	Off	Not Used	60°	Low
S2	On	Off	Not Used	60°	High
S3	On	Off	Not Used	30°	Low
S4	On	Off	Not Used	30°	High
S5	On	Off	Used	60°	Low
S6	On	Off	Used	60°	High
S7	On	Off	Used	30°	Low
S8	On	Off	Used	30°	High
S9	On	On	Not Used	60°	Low
S10	On	On	Not Used	60°	High
S11	On	On	Not Used	30°	Low
S12	On	On	Not Used	30°	High
S13	On	On	Used	60°	Low
S14	On	On	Used	60°	High
S15	On	On	Used	30°	Low
S16	On	On	Used	30°	High

**Table 2:** Cases for heating mode of the four-way cassette fan coil unit in winter.

Winter Season Case					
Case	Ventilation System	Air Cleaner	Curtain	Four-Way Cassette Fan Coil Unit	
				Flow Discharge Angle	Flow Rate
W1	On	Off	Not Used	60°	Low
W2	On	Off	Not Used	60°	High
W3	On	Off	Not Used	45°	Low
W4	On	Off	Not Used	45°	High
W5	On	Off	Used	60°	Low
W6	On	Off	Used	60°	High
W7	On	Off	Used	45°	Low
W8	On	Off	Used	45°	High
W9	On	On	Not Used	60°	Low
W10	On	On	Not Used	60°	High
W11	On	On	Not Used	45°	Low
W12	On	On	Not Used	45°	High
W13	On	On	Used	60°	Low
W14	On	On	Used	60°	High
W15	On	On	Used	45°	Low
W16	On	On	Used	45°	High

## Data Availability

Not applicable.

## References

[1] Lee S.Y., Choi S.H., Park J.E., Hwang S., Kwon K.T. (2020). Crucial role of temporary airborne infection isolation rooms in an intensive care unit: Containing the COVID-19 outbreak in South Korea. Crit. Care.

[2] Qian H., Miao H., Liu L., Zhen X., Luo D., Li Y. (2021). Indoor transmission of SARS-CoV-2. Indoor Air.

[3] Lu J., Gu J., Li K., Xu C., Su W., Lai Z., Zhou D., Yu C., Xu B., Yang Z. (2020). COVID-19 outbreak associated with air conditioning in restaurant, Guangzhou, China, 2020. Emerg. Infect. Dis..

[4] Baumgarte S., Hartkopf F., Holzer M., von Kleist M., Neitz S., Kriegel M., Bollongino K. (2022). Investigation of a limited but explosive COVID-19 outbreak in a German secondary school. Viruses.

[5] Tang L., Liu M., Ren B., Chen J., Liu X., Wu X., Huang W., Tian J. (2022). Transmission in home environment associated with the second wave of COVID-19 pandemic in India. Environ. Res..

[6] Siegel J.D., Rhinehart E., Jackson M., Chiarello L. (2007). 2007 guideline for isolation precautions: Preventing transmission of infectious agents in health care settings. Am. J. Infect. Control.

[7] Zhu N., Zhang D., Wang W., Li X., Yang B., Song J., Zhao X., Huang B., Shi W., Lu R. (2020). A novel coronavirus from patients with pneumonia in China, 2019. N. Engl. J. Med..

[8] Van Doremalen N., Bushmaker T., Morris D.H., Holbrook M.G., Gamble A., Williamson B.N., Tamin A., Harcourt J.L., Thornburg N.J., Gerber S.I. (2020). Aerosol and surface stability of SARS-CoV-2 as compared with SARS-CoV-1. N. Engl. J. Med..

[9] World Health Organization (2020). Transmission of SARS-CoV-2: Implications for Infection Prevention Precautions. https://www.who.int/news-room/commentaries/detail/transmission-of-sars-cov-2-implications-for-infection-prevention-precautions.

[10] Fears A.C., Klimstra W.B., Duprex P., Hartman A., Weaver S.C., Plante K.S., Mirchandani D., Plante J.A., Aguilar P.V., Fernandeez D. (2020). Persistence of sever acute respiratory syndrome coronavirus 2 in aerosol suspension. Emerg. Infect. Dis..

[11] Setti L., Passarini F., Gennaro G.D., Barbieri P., Perrone M.G., Borelli M., Palmisani J., Gilio A.D., Torboli V., Fontana F. (2020). SARS-CoV-2RNA found on particulate matter of Bergamo in Norhern Italy: First evidence. Environ. Res..

[12] Yao Y., Pan J., Wang W., Liu Z., Kan H., Qiu Y., Meng X., Wang W. (2020). Association of particulate matter pollution and case fatality rate of COVID-19 in 49 Chinese cities. Sci. Total Environ..

[13] Somsen G.A., van Rijin C., Kooij S., Bem R.A., Bonn D. (2020). Small droplet aerosols in poorly ventilated spaces and SARS-CoV-2 transmission. Lancet Respir. Med..

[14] Elsaid A.M., Ahmed M.S. (2021). Indoor air quality strategies for air-conditioning and ventilation systems with the spread of the global coronavirus (COVID-19) epidemic: Improvements and recommendations. Environ. Res..

[15] Srivastava S., Zhao X., Manay A., Chen Q. (2021). Effective ventilation and air disinfection system for reducing coronavirus disease 2019 (COVID-19) infection risk in office buildings. Sustain. Cities Soc..

[16] Dai H., Zhao B. (2020). Association of the infection probability of COVID-19 with ventilation rates in confined spaces. Build. Simul..

[17] Noh J.H., Lee J., Noh K.C., Kim Y.W., Yook S.J. (2018). Effects of hospital ward curtains on ventilation in a four-bed hospital ward. Aerosol Air Qual. Res..

[18] Noh K.C., Yook S.J. (2016). Evaluation of clean air delivery rates and operating cost effectiveness for room air cleaner and ventilation system in a small lecture room. Energy Build..

[19] Casagrande D., Piller M. (2020). Conflicting effects of a portable ultra-clean airflow unit on the sterility of operating rooms: A numerical investigation. Build. Environ..

[20] Liu J., Lin Z. (2020). Performance of stratum ventilated heating for sleeping environment. Build. Environ..

[21] Yin H., Li Y., Deng X., Han Y., Wang L., Yang B., Li A. (2020). Performance evaluation of three attached ventilation scenarios for tiny sleeping spaces. Build. Environ..

[22] Hormigos-Jimenez S., Padilla-Marcos M.A., Meiss A., Gonzalez-Lezcano R.A., Feijo-Munoz J. (2018). Computational fluid dynamics evaluation of the furniture arrangement for ventilation efficiency. Build. Serv. Eng. Res. Technol..

[23] Akbari V., Salmanzadeh M. (2019). Numerical evaluation of the effect of air distribution system and location on performance of a portable air cleaner. Sci. Technol. Built Environ..

[24] Rabanillo-Herrero M., Padilla-Marcos M.A., Feijo-Munoz J., Gil-Valverde R., Meiss A. (2020). Ventilation efficency assessment according to the variation of opening position in L-shaped rooms. Build. Simul..

[25] Navaratnam S., Nguyen K., Selvaranjan K., Zhang G., Mendis P., Aye L. (2022). Designing post COVID-19 buildings: Approaches for achieving healthy buildings. Buildings.

[26] Meiss A., Feijo-Munoz J., Garcia-Fuenes M.A. (2013). Age-of-the-air in rooms according to the environmental condition of temperature: A case study. Energy Build..

[27] Ishiguro R., Chikamoto T., Hashimoto S., Inada R., Nishino A., Akimoto T. (2011). Airflow control for personal air-conditioning in a partly unoccupied zone using a multi-flow ceiling cassette type packaged air-conditioner. Int. J. Vent..

[28] Kato S., Yang J.H. (2008). Study on inhaled air quality in a personal air-conditioning environment using new scales of ventilation efficiency. Build. Environ..

[29] Lee S., Lee J., Kato S. (2017). Influence of vane angle on the effectiveness of air conditioning of wall-mounted split-type air conditioners in residential buildings. Sci. Technol. Built Environ..

[30] Lee J., Noh J.H., Noh K.C., Kim Y.W., Yook S.J. (2021). Effect of a system air conditioner on local air quality in a four-bed ward. Aerosol Air Qual. Res..

[31] Mei S.J., Yuan C. (2021). Analytical and numerical study on transient urban street air warming induced by anthropogenic heat emission. Energy Build..

[32] Chew L.W., Glicksman L.R., Norford L.K. (2018). Buoyant flows in street canyons: Comparison of RANS and LES at reduced and full scales. Build. Environ..

[33] Zhao Y., Chew L.W., Kubilay A., Carmeliet J. (2020). Isothermal and non-isothermal flow in street canyons: A review from theoretical, experimental and numerical perspectives. Build. Environ..

